# Tris-(2-pyridyl)-pyrazolyl Borate Zinc(II) Complexes: Synthesis, DNA/Protein Binding and In Vitro Cytotoxicity Studies

**DOI:** 10.3390/molecules26237341

**Published:** 2021-12-03

**Authors:** Manmath Narwane, Dorothy Priyanka Dorairaj, Yu-Lun Chang, Ramasamy Karvembu, Yu-Han Huang, Hsueh-Wei Chang, Sodio C. N. Hsu

**Affiliations:** 1 Drug Development and Value Creation Research Centre, Department of Medicinal and Applied Chemistry, Kaohsiung Medical University, Kaohsiung 80708, Taiwan; manmathnarwane@gmail.com (M.N.); dorothypriyanka@gmail.com (D.P.D.); alan.y.l.chang66@gmail.com (Y.-L.C.); 2 Department of Chemistry, National Institute of Technology, Tiruchirappalli 620015, India; kar@nitt.edu; 3 Graduate Institute of Medicine, College of Medicine, Kaohsiung Medical University, Kaohsiung 80708, Taiwan; ashleyasline@gmail.com; 4 Department of Biomedical Science and Environment Biology, Kaohsiung Medical University, Kaohsiung 80708, Taiwan; 5 Department of Medical Research, Kaohsiung Medical University Hospital, Kaohsiung 80708, Taiwan

**Keywords:** tris(pyrazolyl)borates, zinc(II), biomolecular interactions, in vitro cytotoxicity, triple negative breast cancer cells

## Abstract

Zn(II) complexes bearing tris[3-(2-pyridyl)-pyrazolyl] borate (Tp^py^) ligand (**1**–**3**) was synthesized and examined by spectroscopic and analytical tools. Mononuclear [Tp^py^ZnCl] (**1**) has a Zn(II) centre with one arm (pyrazolyl-pyridyl) dangling outside the coordination sphere which is a novel finding in Tp^py^Zn(II) chemistry. In complex [Tp^py^Zn(H_2_O)][BF_4_] (**2**) hydrogen bonding interaction of aqua moiety stabilizes the dangling arm. In addition, solution state behaviour of complex **1** confirms the tridentate binding mode and reactivity studies show the exogenous axial substituents used to form the [Tp^py^ZnN_3_] (**3**). The complexes (**1**–**3**) were tested for their ability to bind with Calf thymus (CT) DNA and Bovine serum albumin (BSA) wherein they revealed to exhibit good binding constant values with both the biomolecules in the order of 10^4^–10^5^ M^−1^. The intercalative binding mode with CT DNA was confirmed from the UV-Visible absorption, viscosity, and ethidium bromide (EB) DNA displacement studies. Further, the complexes were tested for in vitro cytotoxic ability on four triple-negative breast cancer (TNBC) cell lines (MDA-MB-231, MDA-MB-468, HCC1937, and Hs 578T). All three complexes (**1**–**3**) exhibited good IC_50_ values (6.81 to 16.87 μM for 24 h as seen from the MTS assay) results which indicated that these complexes were found to be potential anticancer agents against the TNBC cells.

## 1. Introduction

Zinc is the second most abundant metal found in the human body (human being contains average 2–3 g of zinc), being vital in regulating cellular process [1,2] including enzyme regulation [3], gene expression [4], apoptosis [5], and neurotransmission [6]. Compared to other first row transition metals, zinc has certain distinguishing properties which makes it quite challenging in the biological domain [3,7]. The fast exchange with ligands, strong Lewis’s acidity, redox inactiveness, and the capability to endure a versatile coordination geometry are some of the striking features which makes zinc ubiquitous in the biological system [8]. It is a widely known fact that Zn(II) ions are redox inert in biology [9], where zinc always remains in the Zn(II) valence state and can neither oxidize nor reduce another substance [10]. The zinc and the cysteine–sulfur bond present in the proteins play key roles in controlling cellular processes, maintaining homeostasis, cellular signaling, enzyme catalysis, and also influencing cytotoxicity [11]. With the above mentioned redox-inert characteristic properties, we find that zinc is functionally closer to the redox-inert magnesium and calcium ions and among the redox-inert divalent metal ions, zinc is the metal that has the strongest interaction with ligand frameworks and biomolecules [12].

*N*-donor ligands are considered to be an important class of ligand system due to their intrinsic toxicity and DNA intercalation properties. For example, 2,2′-bipyridine, terpyridines, 1,10-phenanthrolines, planar aromatic quinolines, and pyrazoles based ligands showed promising antineoplastic activity when combined with first row transition metals [13,14,15,16]. Changing the ligands environment towards the specific target is one of the ways in tuning the selectivity of the drug molecule [17]. The nature of the ligand is expected to play an important role in binding with metal complexes as well as biomolecules [18]. Apart from the biological activity, the pyrazole-based ligands framework are also utilized in the field of drug delivery in the form of metal organic frameworks and coordination cages [19,20]. One such ligand system that assembled in a cage manner is the anionic tris[3-(2-pyridyl)-pyrazolyl]borate (Tp^py^) which possesses a hexadentate cavity suitable for lanthanides and actinides to form mononuclear luminescent and fluorescent complexes [21]. On the other hand, the hexadentate anionic [Tp^py^]^−^ ligand with first row transition metals may result in dinuclear and trinuclear complexes with copper(I) and tetranuclear complexes with manganese(II) or zinc(II) metals [22,23]. The polydentate ligands usually resulted into the self-assembled products which are controlled either by ligand geometry or ligand field stabilization energy (LFSE) of metal complexes. Notably, these first-row transition metals with their chloride salts and anionic [Tp^py^]^−^ ligand may bind in a distorted square pyramidal arrangement with two bidentate pz^py^ arms. Due to the axial chloride and planarity of bidentate (pz^py^) arms, one bidentate arm unit fails to coordinate to the same metal center. Recently, we investigated the formation of iron(II) complexes with Tp^py^ and explored the coordination behaviors with different sources of iron salt [24].

Combining the biological importance of the Tp^py^ ligand and the zinc metal ion, herein we have synthesized three Zn(II) complexes bearing different substitutions and their structure has been confirmed from the single crystal X-ray diffraction study. In addition, we have emphasized on the solid state and solution state behavior of the complexes through FT-IR and NMR spectroscopic methods. The complexes have been evaluated for their DNA and protein (BSA) binding efficacy and are also tested for their in vitro cytotoxic ability on four triple-negative breast cancer cell lines namely, MDA-MB-231, MDA-MB-468, HCC1937, and Hs 578T.

## 2. Materials and Methods

General procedures, chemical reagents, and X-ray crystal determination details are provided in the supporting information.

### 2.1. Complex Synthesis

#### 2.1.1. [Tp^py^ZnCl] (**1**)

A solution of KTp^py^ (0.200 g, 0.410 mmol) in 10 mL of methanol was added to a solution of anhydrous ZnCl_2_ (0.055 g, 0.410 mmol) in 5 mL of methanol upon which a white precipitate formed immediately. The mixture was stirred for 2 h and solvent evaporated to one third and filtered. The resulting product was washed with methanol (5 mL) and dichloromethane (5 mL) which was then dried under vacuum. Yield: 0.172 g (77%). X-ray quality crystals were grown from methanol solution at −20 °C. Anal. Calculated for C_24_H_19_BN_9_ZnCl: C, 52.88; H, 3.51; N, 23.13. Found: C, 52.92; H, 3.50; N, 23.15. UV-Vis [DMSO; λ_max_/nm (ε/dm^3^mol^−1^cm^−1^)]: 285(11,400), FT-IR (KBr, cm^−1^): ν_B-H_ 2440(w). ^1^H NMR (400 MHz, CDCl_3_, δ): 8.76 (s, 1H), 7.81 (s, 3H), 7.34 (s, 1H), and 6.69 (s, 1H). ESI-MS (+): 507.98 [Tp^py^Zn]^+^.

#### 2.1.2. [Tp^py^Zn(H_2_O)](BF_4_)] (**2**)

A solution of KTp^py^ (0.200 g, 0.410 mmol) in 10 mL of methanol was added to a solution of [Zn(H_2_O)_6_](BF_4_)_2_ (0.098 g, 0.410 mmol) in 5 mL of methanol upon which a clear solution formed immediately. The mixture was stirred for 2 h and solvent evaporated. The product was dissolved in dichloromethane (40 mL) and filtered through celite. The solvent was evaporated to one-third yielding a white crystalline compound. Yield: 0.170 g (77%). X-ray quality crystals were grown from methanol solution at −20 °C. Anal. Calculated for C_24_H_21_B_2_N_9_OZnF_4_: C, 54.63; H, 4.01; N, 23.89. Found: C, 54.65; H, 4.03; N, 23.85. UV-Vis [DMSO; λ_max_/nm (ε/dm^3^mol^−1^cm^−1^)]: 287(11,480), FT-IR (KBr, cm^−1^): ν_B-H_ 2480(w). ^1^H NMR (400 MHz, CDCl_3_, δ): 9.05 (s, 1H), 8.12 (s, 1H), 7.95 (s, 2H), and 6.90 (s, 1H). ESI-MS(+): 507.98 [Tp^py^Zn]^+^.

#### 2.1.3. [Tp^py^ZnN_3_] (**3**)

A solution of **1** (0.100 g, 0.184 mmol) in 10 mL of methanol was added to a solution of NaN_3_ (0.012 g, 0.184 mmol) in 5 mL of methanol. After completion of the reaction, the solvent was evaporated to give off a white solid after 2 h. Re-dissolving this solid in 30 mL of dichloromethane which was then filtered through celite gave a colorless solution. The slow evaporation of this solution resulted in an off-white product. Yield: 0.060 g (59%). X-ray crystals were obtained by the slow diffusion of hexane in dichloromethane solution. Anal. Calculated for C_24_H_19_BN_12_Zn: C, 52.25; H, 3.47; N, 30.47. Found: C, 52.28; H, 3.49; N, 30.50. UV-Vis [DMSO; λ_max_/nm (ε/dm^3^mol^−1^cm^−1^)]: 290(11,600), FT-IR (KBr, cm^−1^): ν_B-H_ 2425(w), ν_N3_ 2092 (s). ^1^H NMR (400 MHz, CDCl_3_, δ): 8.75 (s, 1H), 7.89 (s, 3H), 7.37 (s, 1H), and 6.68 (s, 1H). ESI-MS (+): 507.98 [Tp^py^Zn]^+^.

### 2.2. CT DNA Interaction Study

The interaction of the complexes with CT DNA was carried out in Tris-HCl/NaCl buffer maintained at a pH of 7.2. The bulk solution of CT DNA was prepared using Tris-HCl buffer and stored at 4 °C for not more than a week. The CT DNA stock solution gave a UV absorbance ratio of 1.89 at 260 and 280 nm (A260/A280) [25], which indicated that the DNA solution was free of proteins. The concentration of the nucleic acid solutions was determined by UV absorbance at 260 nm after 1:100 dilutions, and the corresponding extinction coefficient at this absorption was 6600 M^−1^cm^−1^. The concentration of CT DNA here was expressed in terms of DNA base pairs. Test solutions of the zinc complexes were prepared using 5% DMF/Tris-HCl/NaCl. The absorption titrations were performed by varying the CT DNA concentration (0–45 µM) against the fixed concentration of the complexes (20 µM).

The complexes did not emit fluorescence. As a result of which, EB displacement study has been employed using fluorescence spectroscopic technique to examine whether the complexes could displace ethidium bromide (EB) from its CT DNA-EB complex. The EB solution was prepared using Tris-HCl buffer (pH 7.2). The complexes (0–50 µM) were titrated with DNA-EB (5 µM), and the corresponding change in fluorescence intensity at 610 nm (excitation wavelength = 450 nm) was noted down.

### 2.3. Hydrodynamic Study

Viscosity experiments were performed on a Micro-Ubbelohde viscometer from the Xylem brand company of German make placed in a thermostatic water bath at 27 °C. The DNA concentration was fixed at 100 µM, while the complex concentration varied from 0 to 60 µM. Flow time was measured three times for each addition, and the average flow time was calculated. The values of relative specific viscosity (η/η_0_)^1/3^, where η is the relative viscosity of DNA in the presence of complex, and η_0_ is the relative viscosity of DNA alone, were plotted against 1/R (1/R = [compound]/[DNA]). Relative viscosity (η_0_) values were calculated from the observed flow time of the DNA solution (t) corrected for the flow time of the buffer alone (t_0_), using the expression η_0_ = (t − t_0_)/t_0_ [26].

### 2.4. Interaction of the Complexes with BSA

The protein binding of the zinc complexes was studied by UV-visible and fluorescence quenching experiments. The excitation wavelength was fixed at 280 nm, and the emission signals at 345 nm were recorded upon the addition of complex to BSA. The stock solution of BSA was prepared using 50 mM phosphate buffer (pH 7.2) and stored at 4 °C for the subsequent use. The stock solutions of the complexes were prepared by dissolving them in DMF-phosphate buffer (5:95) and diluted further with phosphate buffer to obtain the required concentrations. The excitation and emission slit widths, and scan rate were maintained constant throughout the course of the experiment.

### 2.5. Molecular Docking Study with DNA

The molecular docking studies were carried out using Autodock. The compounds were prepared from their 2D chemical structure whose structures were minimized in Gaussian and adding the polar hydrogens, and partial charges, and defining the rotatable bonds that are explored during the docking. The three-dimensional structural coordinates of CT DNA were downloaded from Protein Data Bank (PDB id:1BNA). The Autodock parameters for boron and zinc were added to the parameter input file. The grid box was set with x,y,z coordinates as (60 Å, 60 Å, 87 Å) with a grid spacing of 0.45 Å. The ligands were treated flexibly and the receptor was kept rigid in all the three cases. The genetic algorithm implemented in Autodock was utilized for all the three compounds for systematic optimization of molecular poses. The results were analyzed from the docking log files (dlg) files and the docked conformations with least binding free energy were chosen for each ligand to generate the pictures.

### 2.6. In Vitro Cytotoxicity Study

Cell viability was measured after 24 h drug treatments by the MTS [3-(4,5-dimethylthiazol-2-yl)-5-(3-carboxymethoxyphenyl)-2-(4-sulfophenyl)-2H-tetrazolium] assay [27]. All the TNBC cell lines were procured from American Type Culture Collection (ATCC; Manassas, VA, USA. According to user manual, an MTS [3-(4,5-dimethylthiazol-2-yl)-5-(3-carboxymethoxyphenyl)-2-(4-sulfophenyl)-2H-tetrazolium] assay (Promega; Madison, WI, USA) was performed to evaluate the inhibitory concentration (IC_50_) of the complexes in the TNBC cell lines. Using an ELISA multi-well plate reader purchased from BioTek Instruments, Inc., Winooski, VT, USA, the optical density was measured at 490 nm. The results were used to calculate the percentage (%) of viability using the formula [28], % of viability = (OD value of experimental sample/OD value of experimental control) × 100.

## 3. Results and Discussion

### 3.1. Synthesis and Structure of Zinc Complexes

**Scheme 1:**
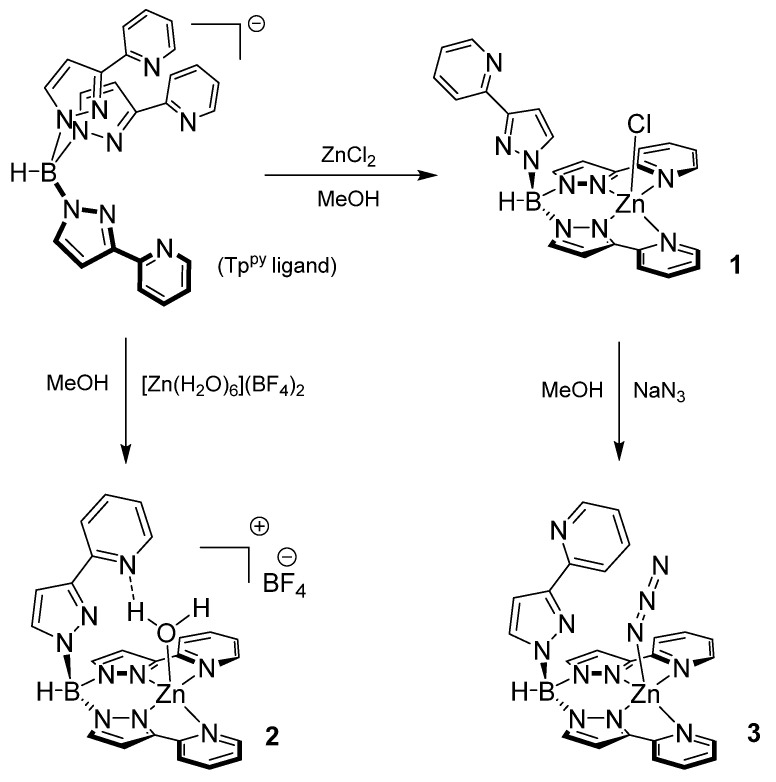
Synthesis of mononuclear Tp^py^Zn(II) complexes.

**Figure 1:**
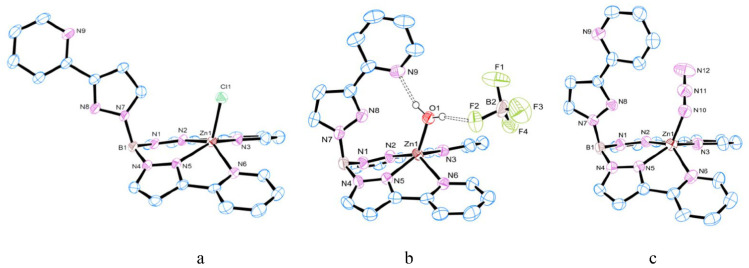
Molecular structure of (**a**) [Tp^py^ZnCl] (**1**), (**b**) [Tp^py^Zn(OH_2_)](BF_4_) (**2**) and (**c**) [Tp^py^Zn(N_3_)] (**3**), showing 50% ellipsoid probability and partial atom numbering schemes. All hydrogen atoms were omitted for clarity except those of water molecule in complex **2**.

An equimolar amount of ZnCl_2_ and [Tp^py^]^−^ in a methanolic solution provides a white powder of mononuclear complex [Tp^py^ZnCl] (**1**) (Scheme 1). An X-ray quality crystal for complex **1** was obtained in methanol at room temperature. Distorted square pyramidal geometry with coordination of the two pz^py^ arms of Tp^py^ ligand and axial ligation of chloride anion, while the third pz^py^ arm of Tp^py^ is dangling out of the coordination sphere confirms the molecular structure of **1** (Figure 1a). The rigidity of the basal plane does not allow the coordination of third arm, and it remains uncoordinated and dangling out with an axial chloride ion. Formation Tp^py^Zn(OH_2_)](BF_4_) (**2**) involves an equivalent amounts of [Zn(H_2_O)_6_](BF_4_)_2_ and [Tp^py^]^−^ ligand in methanol and recrystallization in a dichloromethane at −20 °C gives a single X-ray quality crystal (Scheme 1). The crystallographic data and molecular geometry for **2** is shown in Figure 1b and the corresponding refinement parameters, bond length and bond angle are given in Tables S1 and S2. Complex **2** has a similar geometry like complex **1** with an axial water molecule depicting the hydrogen bonding interaction with the dangling bidentate pz^py^ arm. Similar crystallographic results of copper analogous were reported [29]. It is interesting to note that, effective space management around the coordinated metal in tris-pyrazolyl borates can be achieved through regiospecific placement of selected substituents in the pyrazolyl 3-poisition. Usually these substituents either result as M(Tp)_2_ octahedral with symmetrical binding mode [22,30,31,32,33] or TpM–X tetrahedral with tridentate binding mode. In zinc, [Tp^py^]^−^ with zinc acetate results in the formation of a tetramer in which each of the bidentate arms binds to the three zinc metal ion forming a self-assembled tetrameric complex [23]. However, changing metals salt source and solvent may drive the formation of mononuclear complex. The Zn–O bond distance (1.952(**3**) Å) of **2** is significantly longer than those reported for Zn–OH complexes [34], but similar distance found in the penta-coordinated zinc aqua complexes (1.901–1.982 Å) [35,36,37]. An inward rotation of pendant Pz^py^ arms of Tp^py^ ligands show the hydrogen bonding interaction with axial water molecule thereby stabilizing the H_2_O ligation. The O(1)…N(6) distance in **2** (2.635(**5**) Å) are well within the donor acceptor atoms participating in the hydrogen bonding interactions. The existence of hydrogen bonding interaction is a clear indication of the acidic nature of the coordinated water molecule in the [Tp^py^Zn(OH_2_)]^+^ cation. Parkin et al. studied these interaction in Tp^tBu,Me^Zn-OH_2_…OHB(C_6_F_5_)_3_, as a species Tp^tBu,Me^Zn(OH) stabilized by hydrogen bond to a base whose corresponding acid is [Tp^tBu,Me^Zn(OH_2_)]^+^ [38,39]. As the existence of hydrogen bonding interaction is clear representation of acidic nature of coordinated water molecule in the [Tp^py^Zn(OH_2_)]^+^ cation. In this regard, it is worthy to note that the zinc aqua ligand as the active site of carbonic anhydrase also participate in the hydrogen bond with Thr-199 [40,41,42]; the hydrogen-bonding interaction within the bidentate arm of Tp^py^ and zinc aqua has analogies to that of enzyme active sites.

### 3.2. Substitution Reaction of Mononuclear Zinc Complexes

Reactivity studies for complex **1** was performed by the substitution reaction of N_3_^−^ and replacement of axial chloride by addition of azide salt to the methanolic solution of **1** to form the mononuclear [Tp^py^Zn(N_3_)] (**3**) (Scheme 1). Colorless crystals suitable for X-ray crystallographic analysis were grown by slow diffusion of a hexane and dichloromethane solvent in a 1:2 ratio (Figure 1c). Complex **3** is isomorphous to that of complex **1** with slight distortion in square pyramidal coordination geometry about the Zn(II) center (Figure 1c; Table S1). The azide ligand arranged almost linearly on the square pyramidal plane. The Zn–N(1) bond distance of 2.061(7) Å is slightly longer than reported zinc azide complexes (2.046 Å) [43,44]. Distance between the N(1) and N(2) 1.189(8) and azide moiety is slightly bend forming angle between Zn(1)–N(1)–N(2) 119.23° manifesting distorted square pyramidal geometry.

### 3.3. Solution Behavior of Zinc Complexes

**Scheme 2:**
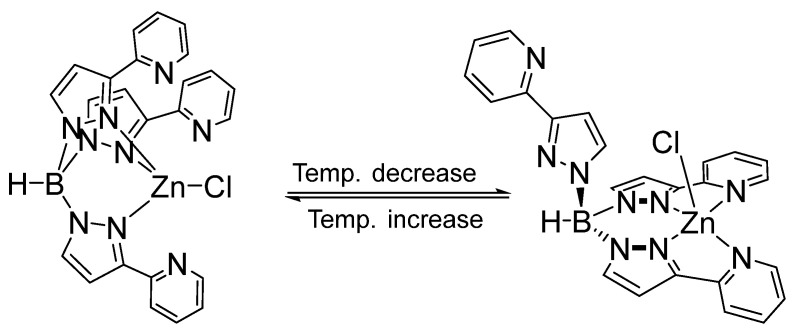
Temperature dependent interconversion of binding mode transformation of **1**.

To understand the solution behavior and gain insight into the dynamic processes of [Tp^py^ZnCl] (**1**) complex, variable temperature ^1^H NMR experiment (VT-^1^H NMR) performed. The main purpose of this study was done to observe the fluxionality taking place in the complexes upon varying the temperature. The VT-^1^H NMR spectrum for complex **1** was recorded from −60 °C to +25 °C in CDCl_3_. Particularly revealing portions of the spectra with assignments for complex **1** are shown in the Figure S1. At 25 °C, a singlet at 8.76 ppm for pyridine (6-CH) proton and other singlet peaks at 7.81, 7.35, and 6.69 ppm was observed which suggested the presence of a [Tp^py^Zn] species. Upon cooling, progressive splitting of the two sets of signals was observed for each singlet, the splitting ratio 2:1 observed upon cooling to −60 °C and dynamic structure of complex may indicate fluxional behavior (as depicted in Scheme 2). Similar observations are also found in the previously reported Tp^4-Py,Me^ and Tp^4-Py^Zn complexes [36,45].

IR spectroscopic studies taken in the solid state reveal the broad absorption peak around 3550 cm^−1^ that characterizes the aqua moiety. The solution state IR studies in dichloromethane illustrates the hydrogen bonding interaction in complex **2** also persist in the solution. The ν_OH_ absorption at 3687, 3601, and 3450 cm^−1^ in the IR spectrum, of which the lowest energy signal at 3450 cm^−1^ is attributed to the hydrogen bonding interaction. However, the hydrogen bond interaction in the solution state for complex **2** is seen only in dichloromethane and not in acetonitrile (Figure S2). Thus, rather than persisting three band patterns associated with the aqua ligand, the IR spectrum possesses two bands that are identical to those of water in acetonitrile.

### 3.4. CT DNA Interaction Study

**Figure 2:**
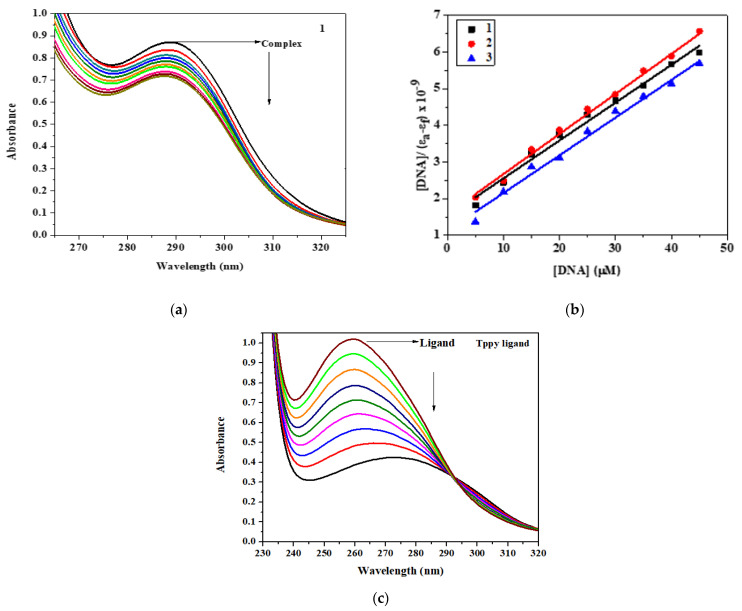
(**a**) Absorption spectra of complex **1** in Tris-HCl buffer upon addition of CT DNA. [complex] = 2.0 × 10^−5^ M, [DNA] = 0–45 µM. The arrow shows that the absorption intensity decreases upon increasing the CT DNA concentration. (**b**) Plot of [DNA]/(ε_a_ − ε_f_) versus [DNA] for the titration of the complexes with CT DNA. (**c**) Absorption spectra of the Tp^py^ ligand in Tris-HCl buffer upon addition of CT DNA. [complex] = 2.5 × 10^−5^ M, [DNA] = 0–45 µM.

Absorption studies are a useful technique deployed to predict the binding efficacy of compounds with CT DNA [46]. Figure 2a and Figure S3 depicts the absorption spectra of the Zn(II) complexes with and without CT DNA. Upon the incremental addition of CT DNA (0–45 µM) to the Zn(II) complexes (20 µM), a uniform decrease in absorbance at 285 nm was seen accompanied by a mild red shift [47]. This hypochromic effect was due to the intercalation of the complexes with DNA [48]. The intrinsic binding constant (*K*_b_) was calculated from the Stern–Volmer equation, [DNA]/(ε_a_ − ε_f_) = [DNA]/(ε_b_ − ε_f_) + 1/*K*_b_ (ε_b_ − ε_f_) [49], where [DNA] is the concentration of DNA in base pairs, ε_a_ is the apparent extinction coefficient value found by calculating A(observed)/[complex], ε_f_ is the extinction coefficient for the free compound, and ε_b_ is the extinction coefficient for the compound in the fully bound form and the corresponding graph is shown in Figure 2. The results showed that all three complexes exhibited a remarkable *K*_b_ value. The binding stoichiometry was such that one molecule of the complex could bind to 3–4 base pairs of CT DNA. The reason could be attributed to the presence of the aromatic pyrazole and pyridine groups inducing planarity in all the complexes causing π–π stacking interactions making them to slide in and penetrate deep in between the DNA base pairs [50,51]. Among them, complex **1** exhibited the highest *K*_b_ value followed by **2** and **3**. Due to the labile nature of the Cl group, on entering the cells gets replaced by OH forming a hydrolysis intermediate which in turn binds strongly to the guanine base pair thus making complex **1** exhibit a higher *K*_b_ value than **2** and **3**. The results were similar to literature reported Zn(II) complexes bearing hexadentate scorpionate type ligands [18]. We also investigated the binding capability of the Tp^py^ ligand with CT DNA (Figure 2c) and found the same decrease in absorbance pattern as that of the Zn(II) complexes. The ligand also adopted an intercalative binding mode with CT DNA, but the intrinsic binding constant (*K*_b_) value was lesser than the *K*_b_ value of the Zn(II) complexes thus highlighting the importance of the zinc metal center in binding with CT DNA.

### 3.5. EB Displacement Study

**Figure 3:**
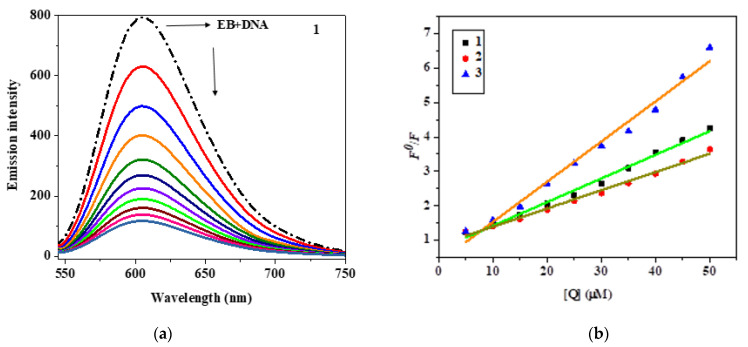
(**a**) Fluorescence quenching curves of EB bound to DNA in the presence of complex **1**. [DNA] = 5 µM, [EB] = 5 µM, and [complex] = 0–50 µM. (**b**) Stern–Volmer plot of fluorescence titrations of the complexes with CT DNA.

To gain more insight on the binding nature of the zinc complexes with CT DNA, the ethidium bromide (EB) displacement study was performed by spectrophotometric studies (Figure 3). As the Zn(II) complexes were non-fluorescent at RT and in the solution state, a competitive study was employed to analyze the DNA-complex binding interaction. As shown in Figure 3a and Figure S4 the emission spectra of the EB-DNA system in the absence and presence of the zinc complexes. On the addition of the complexes (0–50 µM) to EB–DNA, a quenching in the fluorescence intensity at 605 nm was seen. The percentage of hypochromism was 79, 72, and 68 respectively for **1**, **2**, and **3** which revealed the extent of displacement of EB molecules from the study upon addition of the complexes. The extent of quenching (*K*_b_) was calculated from the known Stern–Volmer equation and the apparent DNA binding constant (*K*_app_) was calculated from the formula [52], *K*_EB_ [EB] = *K*_app_ [complex], where [complex] is the complex concentration at 50% reduction in the fluorescence intensity of EB, *K*_EB_ = 1.0 × 10^7^ M^−1^ and [EB] = 5 µM. the order of *K*_q_ and *K*_app_ values for the complexes was **1** > **2** > **3**. The values for *K*_b_, *K*_q_, and *K*_app_ have been enlisted in Table S3 which were in agreement with the CT DNA absorption study results.

### 3.6. Hydrodynamic Study

A precise way of viewing the structural changes taking place in CT DNA is by means of the hydrodynamic study and likewise here the three zinc(II) complexes were subjected to this study. A typical intercalator or an aromatic compound in general tends to slide in between the base pairs of DNA causing breaking of hydrogen bonds in the DNA base pairs thereby lengthening the DNA helix resulting in increased viscosity values [53]. A compound binding to CT DNA via the groove mode causes negligent changes in viscosity values. On the other hand, a non-classical intercalator is said to cause breaking of the DNA helix leading to decreased viscosity values [54]. From Figure S5, we see that upon the incremental addition of the complexes (0–60 µM) to DNA (100 µM), there was a consistent increase in the viscosity value, which suggested intercalation. The order of viscosity values for the complexes is **1** > **2** > **3**, which was in agreement from the results of the titration and fluorescence studies.

### 3.7. Protein Interaction Study of the Zn(II) Complexes with BSA

#### 3.7.1. Absorption Study

Protein-drug interaction plays a key role in chemotherapeutics [55]. In this work, we have chosen BSA as our protein source which is structurally similar to human serum albumin (HSA). The active sites of BSA are Site-I, Site-II and two tryptophan residues namely Trp-134 and Trp-212 [56]. From the absorption spectrum which is shown in Figure S6, an absorption band at 280 nm is seen which is attributed to the π-π* transition belonging to the tryptophan residues [57]. Upon the addition of the complexes (**1**–**3**) to BSA, there was no change in the absorbance value but there was an increase in the absorption intensity(Figures S6 and S7), inferring that a static quenching mechanism on the addition of complexes to BSA took place [58].

#### 3.7.2. Fluorescence Measurement

**Figure 4:**
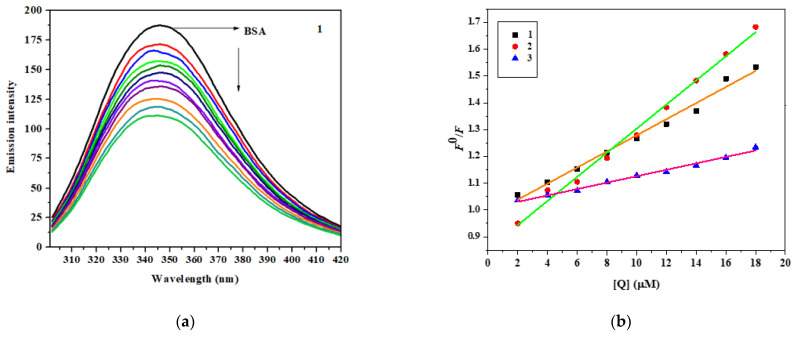
(**a**) a Fluorescence quenching curves of BSA in the absence and presence of **1**. [BSA] = 1 µM and [complex] = 0–20 µM (**b**) Stern–Volmer plot for the fluorescence titrations of complexes **1**–**3** with BSA.

**Figure 5:**
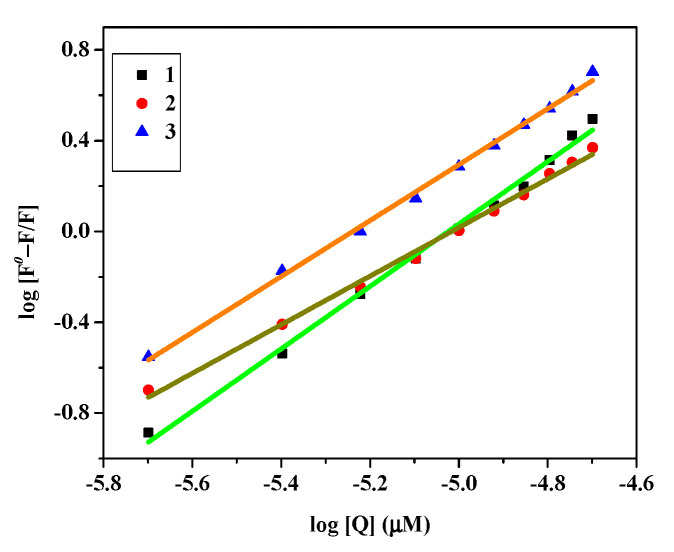
Scatchard plot for the fluorescence titrations of complexes **1**–**3** with BSA.

Fluorescence spectroscopy is a useful tool to understand the mechanism of interaction between the compounds and BSA. Transfer and rearrangement reactions are two important molecular interactions taking place between the target moiety and the quencher that decides the type of quenching to be either static or dynamic. The changes in the fluorescence spectra before and after the addition of the zinc complexes to BSA (Figure 4a and Figure S8). The addition of the complexes (0–20 µM) to BSA (1 µM) lead to a uniform decrease in the fluorescence intensity at 345 nm with quenching percentages of 76, 67, and 64 for **1**–**3** respectively. This quenching mainly resulted due to the active sites of tryptophan resides being buried in a hydrophobic environment and the amount of quenching (*K*_q_) was calculated quantitatively from the Stern–Volmer equation. The plot of *F*^o^/*F* is illustrated in Figure 4b. The equilibrium binding constant (*K*_b_) was calculated from the Scatchard equation [59], log [(*F*^o^ − *F*)/*F*] = log *K*_b_ + *n* log [Q], where *K*_b_ is the binding constant of complex with BSA and *n* is the number of binding sites (Figure 5). From the calculated results, complex **1** exhibited highest *K*_q_ and *K*_b_ values followed by **2** and **3**. The number of binding sites (n) was 1.37, 1.23, and 1.07 respectively which indicated that all the complexes suggested a single occupancy with BSA. The values of *K*_b_, *K*_q_ and *n* are listed in Table S4. In addition, the inner filter effect of the Zn(II) complexes on BSA emission quenching has been corrected using the following equation, F_corr_ = F_obs_ × e^(Aex+Aem)/2^ where F_corr_ is the corrected fluorescence intensity, F_obs_ is the observed intensity. A_ex_ and A_em_ Are the absorbance of the compounds at excitation and emission wavelength, respectively [60]. In the present study, the fluorescence quenching occurring between the Zn(II) complexes and BSA is due to the photoinduced electron transfer (PET) mechanism [61]. When a flow/transfer of electron(s) between BSA and the Zn(II) complexes takes place, the PET process gets inhibited that leads to quenching. The quenching in fluorescence and the enhancement in absorbance upon interaction of the Zn(II) complexes with the tryptophan residues of BSA infers a static quenching mechanism [62].

### 3.8. Molecular Docking Investigation with DNA

**Figure 6:**
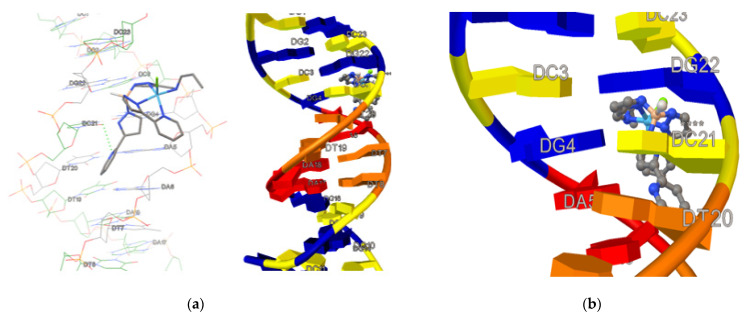
(**a**) Molecular docked pose of **1** with DNA. (**b**) Zoomed image of the molecular docked pose of **1** with DNA.

The docking results of the three Zn(II) complexes were explored for the least binding energy profile of the resultant docking poses. Among the three complexes, **1** binds with DNA with a binding free energy of −10.3 kcal/mol representing the strong binder of DNA whereas the binding free energy values of **2** and **3** are −7.14 and −7.03 kcal/mol. The two pyrazole rings coordinated with zinc of **1** intercalated into DNA in the region stacked by DC21, DG22, DC3, and DG4. A hydrogen bond is also observed between carbonyl part of DC21 and the Pyridine nitrogen which also stabilizes the binding with DNA (Figure 6). **2** was also found to intercalated into DNA as confirmed by experiments in which the coordination site assisted in binding of the compound to DNA (Figure S9). In case of **3**, the pyridine ring coordinated with zinc intercalated into DNA in the same region formed by the base pairs DC21, DG22, and DC3 and DG4 (Figure S10). No hydrogen bonds were found in **2** and **3** and hence the DNA-**2** and DNA-**3** complexes were stabilized by hydrophobic and Van der Waals forces. Thus the docking study revealed the intercalative mode of binding of all the Zn(II) complexes which was also confirmed by the spectral and fluorescence measurements.

### 3.9. In Vitro Cytotoxicity Evaluation of the Zn(II) Complexes: Cell Viability Assay Analysis

In triple-negative breast cancer cells (TNBC) usually common breast cancer markers such as, estrogen receptor (ER), human epidermal growth factor receptor 2 (HER2), and progesterone receptor (PR) are absent and this is one of the main reason for ineffectiveness of common target therapy against TNBC [63]. In the present study, four TNBC cell lines were chosen to demonstrate the cytotoxicity of the complexes **1**, **2**, and **3** after 24 h incubation. Interestingly, all three complexes for 24 h MTS assay showed excellent anticancer activity against all the four cancer cell lines wherein the IC_50_ values ranged from 6.81–10.46 µM on the MDA-MB-231 cells, 8.68–16.56 µM on the MDA-MB-468 cells, 10.67–13.54 µM on HCC1937 cells, and 6.68–16.87 µM on the Hs 578T cancer cells lines, respectively. The IC_50_ values for the complexes against all cell lines are listed in Table 1. For comparison, IC_50_ value for the clinical drug cisplatin was 13.95 µM for MDA-MB-231 cells at 24 h MTS assay [64]. For comparison, IC_50_ value for the clinical drug cisplatin was 32.38 µM for MDA-MB-468 cells at 24 h MTT assay [65]. Therefore, the cytotoxic effects of compound **1**, **2**, and **3** to TNBC cells may have a better performance than cisplatin and the reported N-donor containing zinc complexes [15,16]. However, these Zn-derivative drugs were not tested in the cell viability of normal breast cells. Drug safety for them needs to be concerned before further application. It warrants detailed investigation for the in vitro cytotoxicity of normal breast cells in the future.

## 4. Conclusions

We investigated the reaction of [Tp^py^]^−^ with two different zinc (II) salts to obtain mononuclear Zn(II) (**1**–**2**) complexes. Crystallographic results confirmed the penta-coordinated Zn(II) center with distorted square pyramidal geometries for both complexes. The key geometric differences in complexes **1** and **2** were dangling pyridyl-pyrazole arm. In later case it was stabilized by hydrogen bonding interaction from axial aqua moiety in complex **2**. On the other hand, low temperature NMR data confirms the tetradentate binding mode for the complex **1** in solution state. In addition, the solid and solution state IR for complex **2** shows the disruption of hydrogen bonding interaction in the coordinated solvents. These results indicate the different solid and solution state behavior of these complexes. Further, reactivity of the complex **1** were also tested to obtain axially substituted zinc azide complex **3**. Besides, these complexes were tested for their biological potency by performing the DNA/protein binding study through absorption and spectrophotometric means and also evaluating their cytotoxicity nature by performing the MTS against a panel of four cancer cell lines.

Interestingly, all complexes displayed good binding constant values with both the biomolecules and the mode of binding between the title complexes and DNA was found to be intercalation which was supported by the viscosity study results. The in vitro cytotoxicity results inferred that all the complexes were potent anticancer agents exhibiting excellent activity on all the four cancer cell lines with an IC_50_ value ranging from 6.72 to 16.87 µM owing to the presence of the pyrazole and pyridine units in the synthesized complexes. Therefore, in future we would test the cytotoxicity of the currently synthesized Zn(II) complexes by in vivo method on the MDA-MB-231 and MDA-MB-468 cell lines which would hope to show promising activity.

## Figures and Tables

**Table 1:** IC_50_ (µM) values of the Zn(II) complexes **1**–**3** against TNBC cell lines after 24 h incubation. Data, mean ± SD (*n* = 3).

Cancer Cell Lines	1	2
MDA-MB-231	6.81 ± 0.98	8.85 ± 1.05
MDA-MB-468	16.56 ± 1.32	10.85 ± 1.72
HCC1937	13.54 ± 1.77	10.60 ± 1.04
Hs578T	12.51 ± 1.84	6.68 ± 1.16

## Data Availability

The data is contained within the article and also in the electronic supporting information.

## Supplementary Materials

The following are available online, Figure S1: Variable temperature ^1^H-NMR spectra of the complex **2** in d-chloroform (5.2–9.6 ppm), Figure S2: IR spectra of complex **2** in (a) KBr, (b) dichloromethane solution, and (c) acetonitrile solution. The solid state and in dichloromethane solution indicate that hydrogen bonding interaction is retained and in acetonitrile solution is disrupted, Figure S3: Absorption spectra of the Zn(II) complexes in Tris-HCl buffer upon addition of CT DNA. [complex] = 2.0 × 10^−5^ M, [DNA] = 0–45 µM. The arrow shows that the absorption intensity decreases upon increasing the CT DNA concentration, Figure S4: Fluorescence quenching curves of EB bound to DNA in the presence of Zn(II) complexes. [DNA] = 5 µM, [EB] = 5 µM and [complex] = 0–50 µM, Figure S5: Effect of complexes **1**–**3** on the viscosity of CT DNA, Figure S6: Absorption spectra of BSA (10 µM) and BSA with **1**–**3** (4 µM), Figure S7: UV-Visible absorption spectra of BSA (10 µM) with complex **1**–**3**. Figure S8: Fluorescence quenching curves of BSA in the absence and presence of the complexes. [BSA] = 1 µM and [complex] = 0–20 µM. Figure S9: Molecular docked pose of complex **2** with DNA, Figure S10: Molecular docked pose of complex **3** with DNA. Figures S11–S19: NMR, FT-IR, ESI-Mass spectra for complexes **1**–**3**. Table S1: Selected bond distances (Å) and angles (°) in complexes **1**–**3**, Table S2: Crystallographic data for complexes **1**–**3**, Table S3: DNA binding constant (*K*_b_), quenching constant (*K*_q_) and apparent binding constant (*K*_app_) values, Table S4: Protein binding constant (*K*_b_), quenching constant (*K*_q_) and number of binding sites (*n*) values. The CCDC numbers for the complexes **1**–**3** are 2119667–2119669.

## Sample Availability

Samples of the compounds are available from the authors upon request.
