# Supplementary material for: Tris-(2-pyridyl)-pyrazolyl Borate Zinc(II) Complexes: Synthesis, DNA/Protein Binding and In Vitro Cytotoxicity Studies
Source: Molecules. 2021 Dec 3;26(23):7341. doi: 10.3390/molecules26237341 (PMC8659194; doi:10.3390/molecules26237341)
Supplement: Supplementary file 1 [file molecules-26-07341-s001.zip › molecules-1470940-supplementary.pdf]

Electronic Supporting Information

Tris-(2-Pyridyl)-Pyrazolyl Borate Zinc(II) Complexes: Synthesis, DNA/Protein Binding and *In vitro* Cytotoxicity Studies

Manmath Narwane[a,‡], Dorothy Priyanka Dorairaj[a,b,‡], Yu-Lun Chang[a], Ramasamy Karvembu[b], Yu-Han Huang[c], Hsueh-Wei Chang[d,e]*, Sodio C.N. Hsu[a,e]*

[a] *Department of Medicinal and Applied Chemistry, Drug Development and Value Creation Research Centre, Kaohsiung Medical University, Kaohsiung 80708, Taiwan*

[b] *Department of Chemistry, National Institute of Technology, Tiruchirappalli, 620 015, India*

[c] *Graduate Institute of Medicine, College of Medicine, Kaohsiung Medical University, Kaohsiung 80708, Taiwan*

[d] *Department of Biomedical Science and Environment Biology, Kaohsiung Medical University, Kaohsiung 80708, Taiwan*

[e] *Department of Medical Research, Kaohsiung Medical University Hospital, Kaohsiung 80708, Taiwan.*

‡ Both the authors contributed equally to this work.

1. General Procedure

All manipulations were carried out under an atmosphere of purified dinitrogen with standard Schleck techniques. Chemical reagents were purchased from Aldrich chemical co. Ltd., Lancaster chemical Ltd., or Fluka, Ltd. All the reagents were used without further purification, apart from all solvent that dried over Na (Et_2O, THF) or CaH_2 (CH_2Cl_2, CH_3CN) or Mg and potassium iodide (Methanol) and then thoroughly degassed before use. The KTp^{py} ligand was prepared as described in the literature.[1,2] FT-IR spectra recorded on Bruker ALPHA spectrometer. UV-Vis spectra recorded on Agilent 8453 spectrophotometer. ^{1}H NMR spectra were acquired on a Jeol-400 FT-NMR spectrometer. ESI-MS spectra collected on waters ZQ 4000 mass spectrometers. Elemental analysis performed on a Heraeus CHN-OS rapid element analyzer with 0.3% instrumental error.

2. X-ray Crystal Structure Determination

All single X-ray diffraction data were accumulated using Bruker Nonius Kappa CCD diffractometer with Mo Kα radiation (λ=0.71073 Å). The data collection was executed using the SMART program[3]. Cell refinement and data reduction were made with the SAINT program. The structure was determined using the SHELXTL/PC program[4] and refined using full matrix least squares

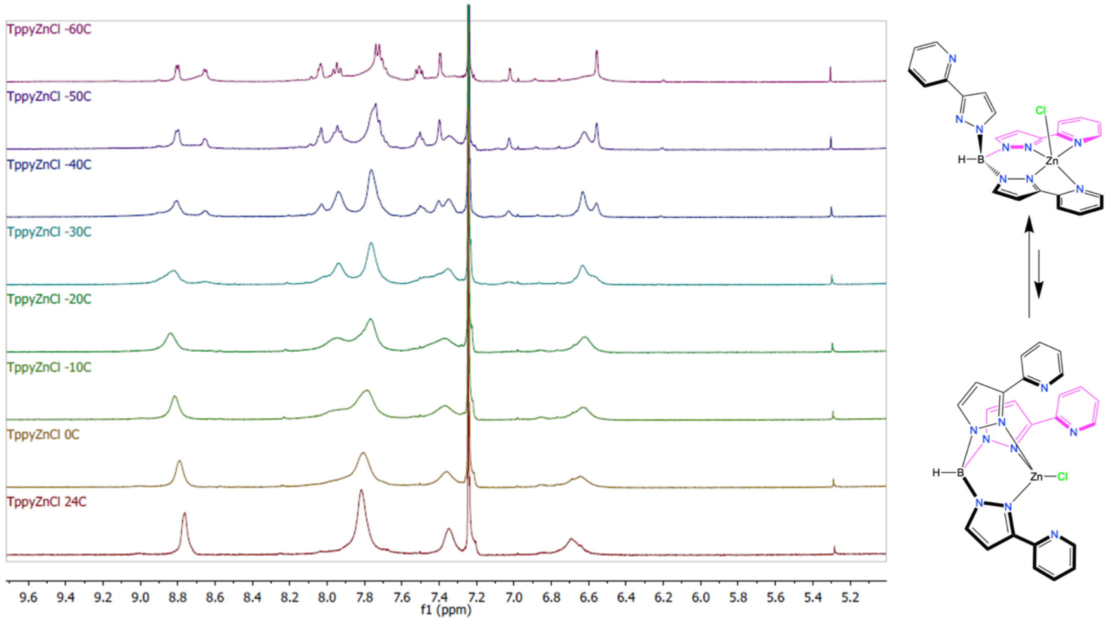

Figure S1. Variable-temperature ^{1}H-NMR spectra of the complex **2** in d-chloroform (5.2-9.6 ppm)

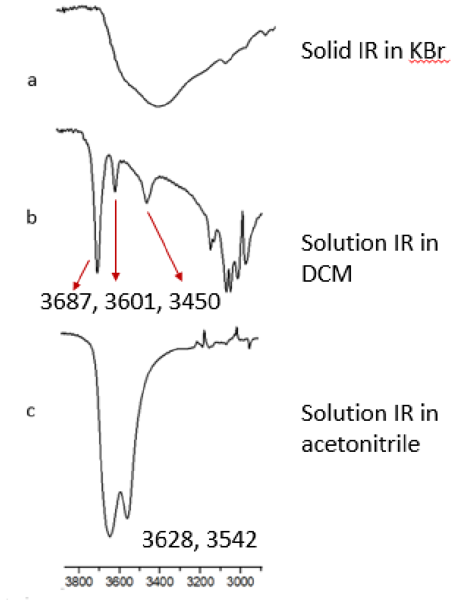

Figure S2. IR spectra of complex **2** in (a) KBr, (b) dichloromethane solution, and (c) acetonitrile solution. The solid state and in dichloromethane solution indicate that hydrogen bonding interaction is retained and in acetonitrile solution is disrupted.

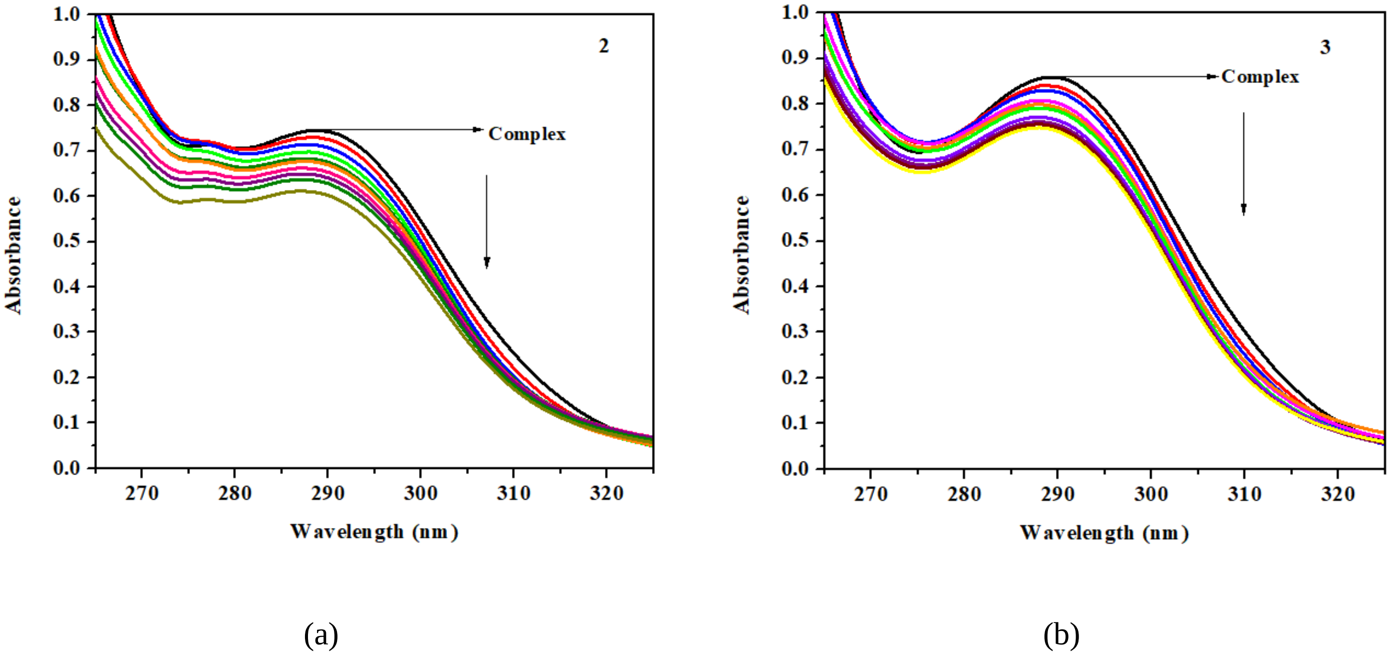

Figure S3. (a) Absorption spectra of complex **2** in Tris-HCl buffer upon addition of CT DNA. [complex] = 2.0 × 10^{-5} M, [DNA] = 0-45 μM. (b) Absorption spectra of complex **3** in Tris-HCl buffer upon addition of CT DNA. [complex] = 2.0 × 10^{-5} M, [DNA] = 0-45 μM. (The arrow shows that the absorption intensity decreases upon increasing the CT DNA concentration).

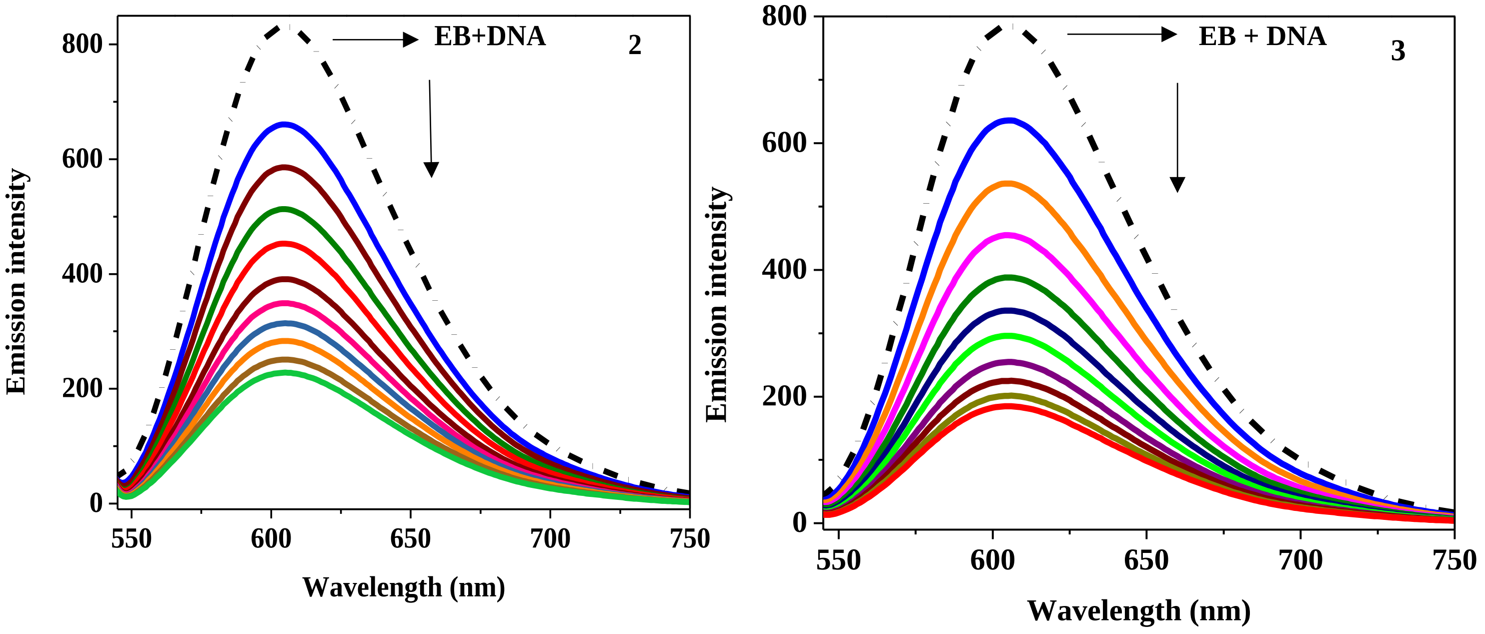

(a) (b)

Figure S4. (a) Fluorescence quenching curves of EB bound to DNA in the presence of complex **2**. [DNA] = 5 µM, [EB] = 5 µM and [complex] = 0-50 µM. (b) Fluorescence quenching curves of EB bound to DNA in the presence of complex **3**. [DNA] = 5 µM, [EB] = 5 µM and [complex] = 0-50 µM. (The downward arrow indicates the quenching in the fluorescence upon addition of the Zn(II) complexes to EB-DNA).

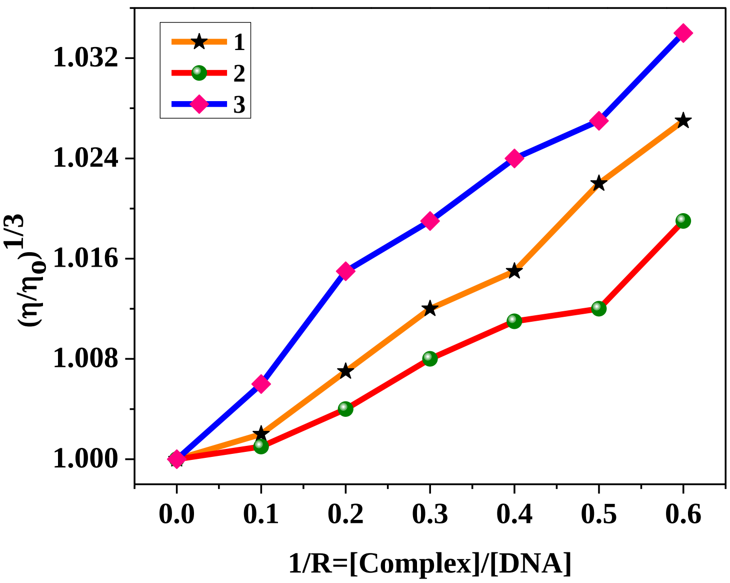

Figure S5. Effect of complexes **1-3** on the viscosity of CT DNA

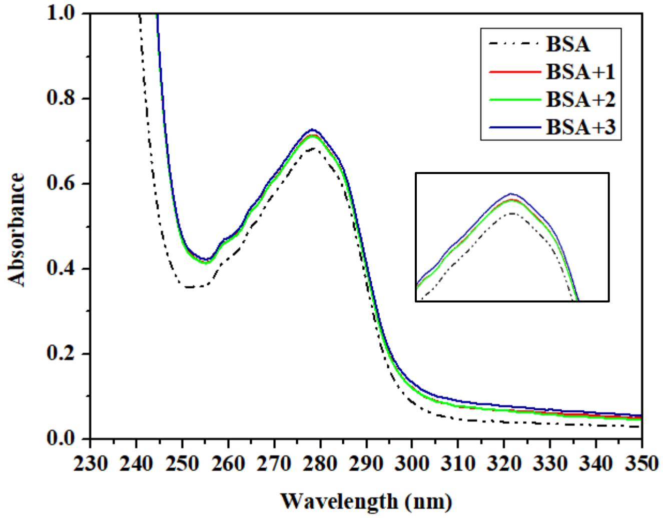

Figure S6. Absorption spectra of BSA (10 µM) and BSA with **1-3** (4 µM).

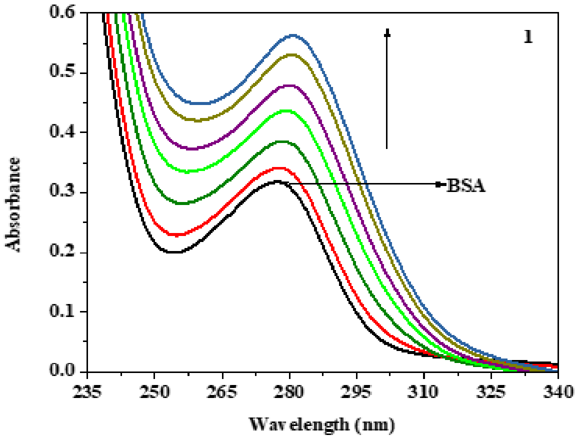

(a)

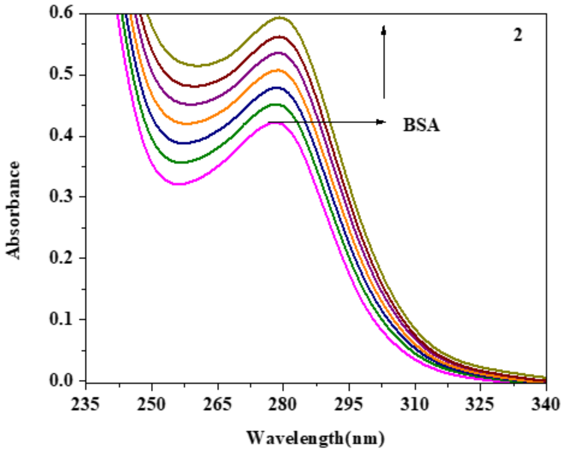

(b)

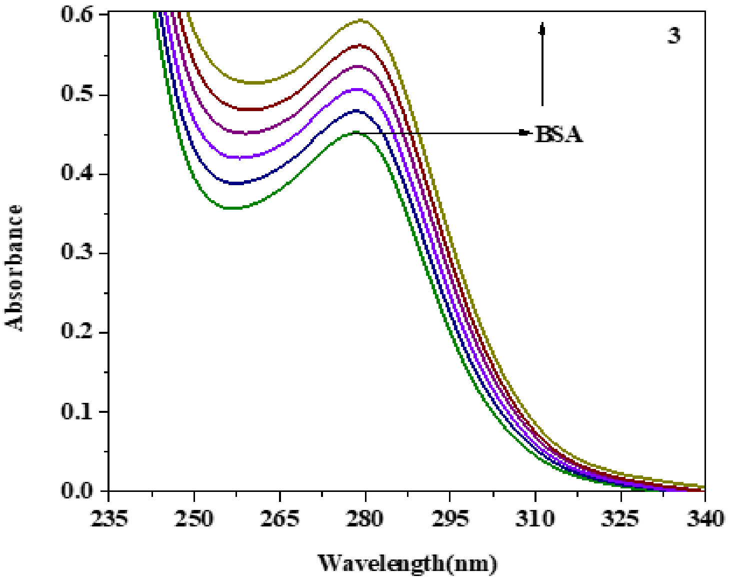

(c)

Figure S7. (a) UV-Visible absorption spectra of BSA (10 µM) with complex **1**(0-30 µM). (b) UV-Visible absorption spectra of BSA (10 µM) with complex **2**(0-30 µM). (c)UV-Visible absorption spectra of BSA (10 µM) with complex **3**(0-30 µM).

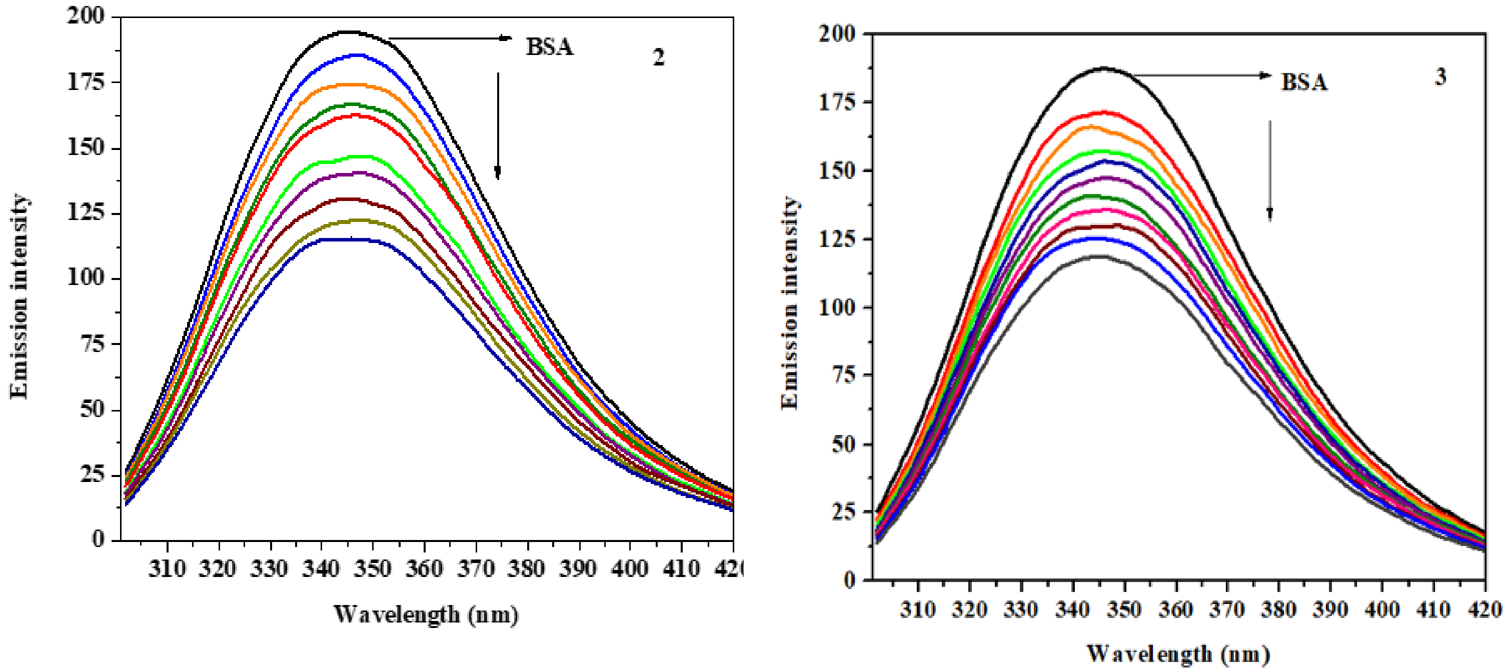

(a) (b)

Figure S8. (a) Fluorescence quenching curves of BSA in the absence and presence of the complex **2**. [BSA] = 1 μM and [complex] = 0-20 μM. (b) Fluorescence quenching curves of BSA in the absence and presence of the complex **3**. [BSA] = 1 μM and [complex] = 0-20 μM. (The downward arrow indicates the quenching in the fluorescence upon addition of the Zn(II) complexes to BSA).

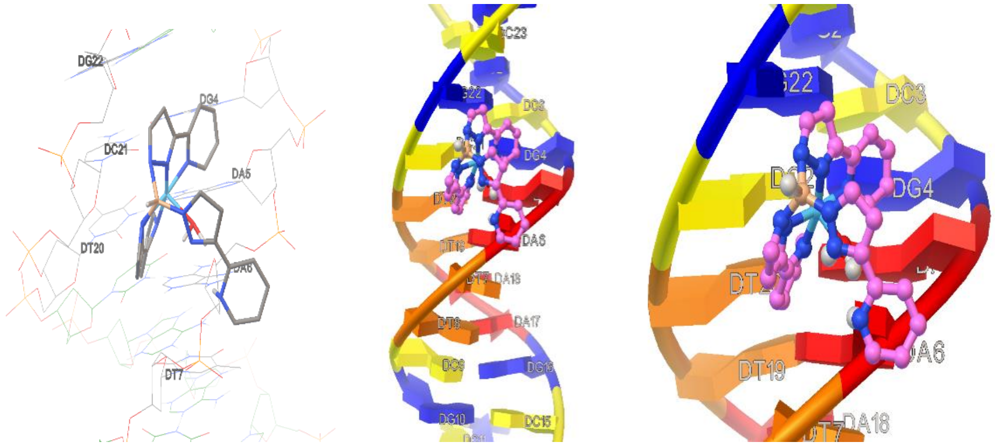

Figure S9. Molecular docked pose of complex **2** with DNA.

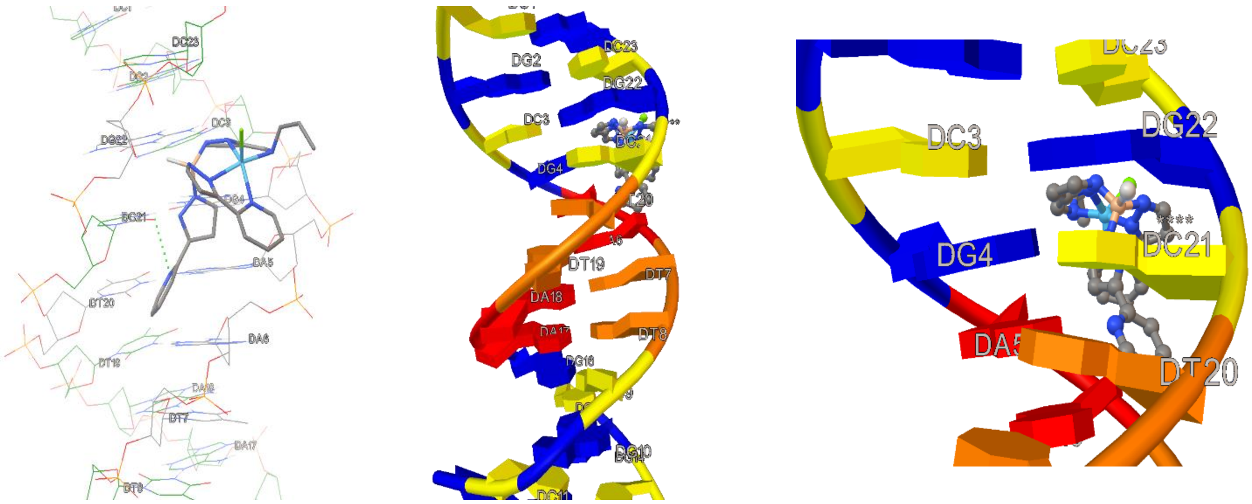

Figure S10. Molecular docked pose of complex **3** with DNA.

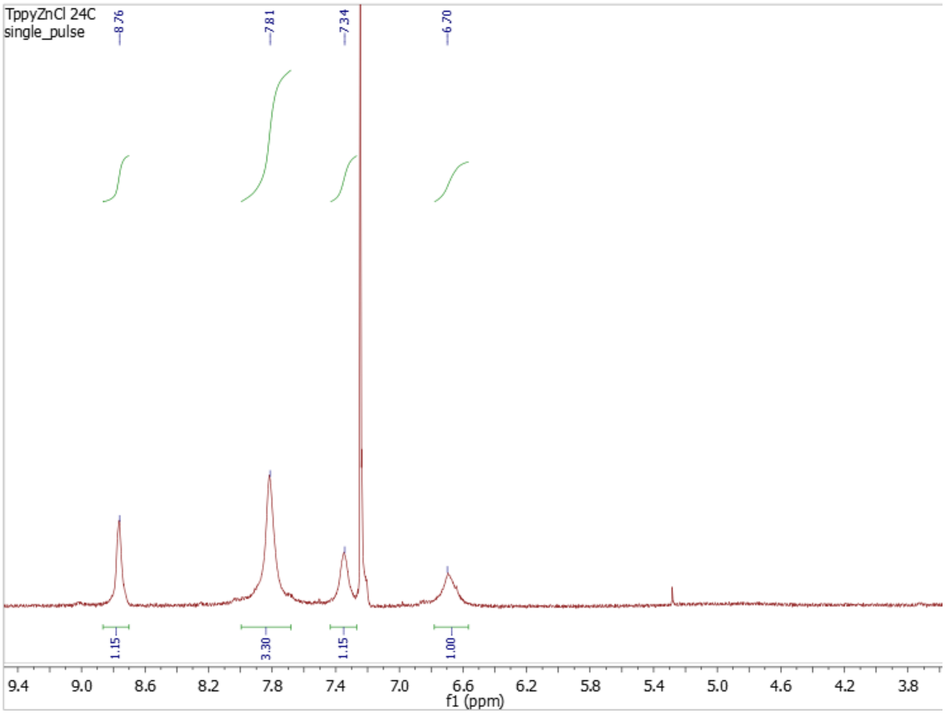

Figure S11. H^1-NMR spectrum for complex **1** in d-chloroform.

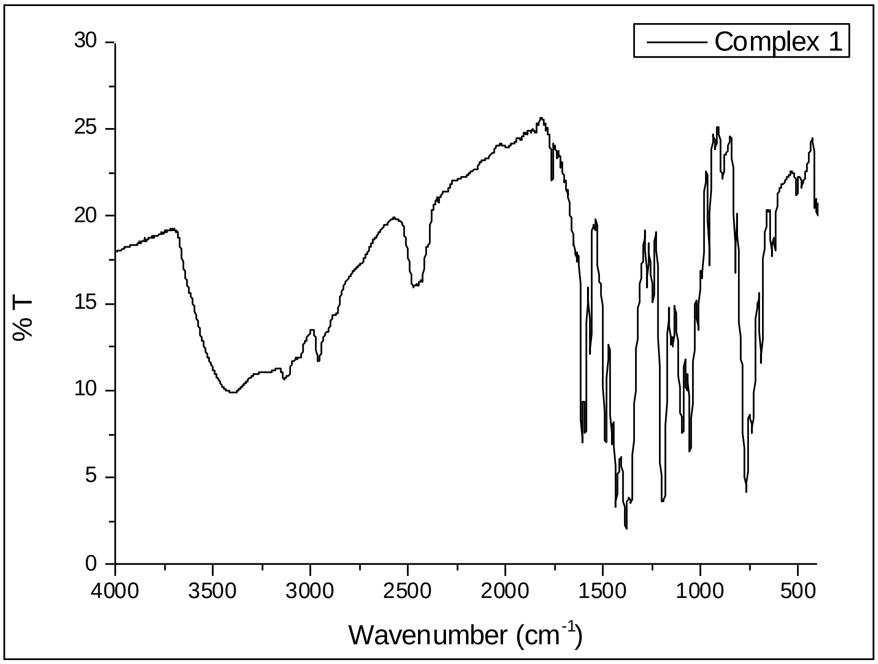

Figure S12. Solid IR spectra for complex **1**

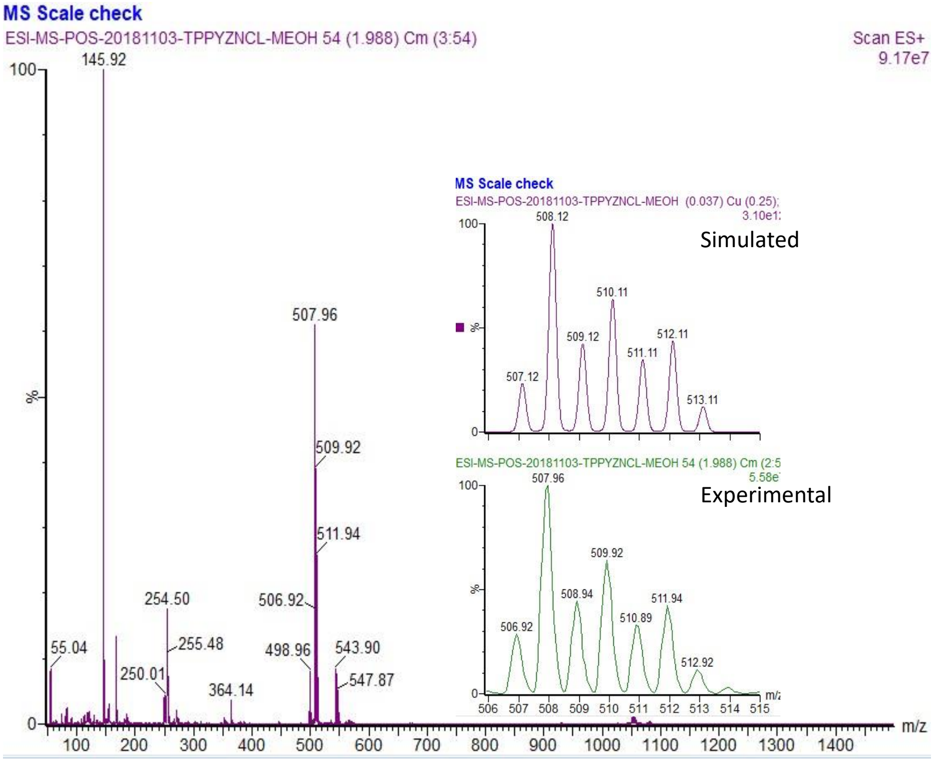

Figure S13. ESI-Mass spectra for complex **1**

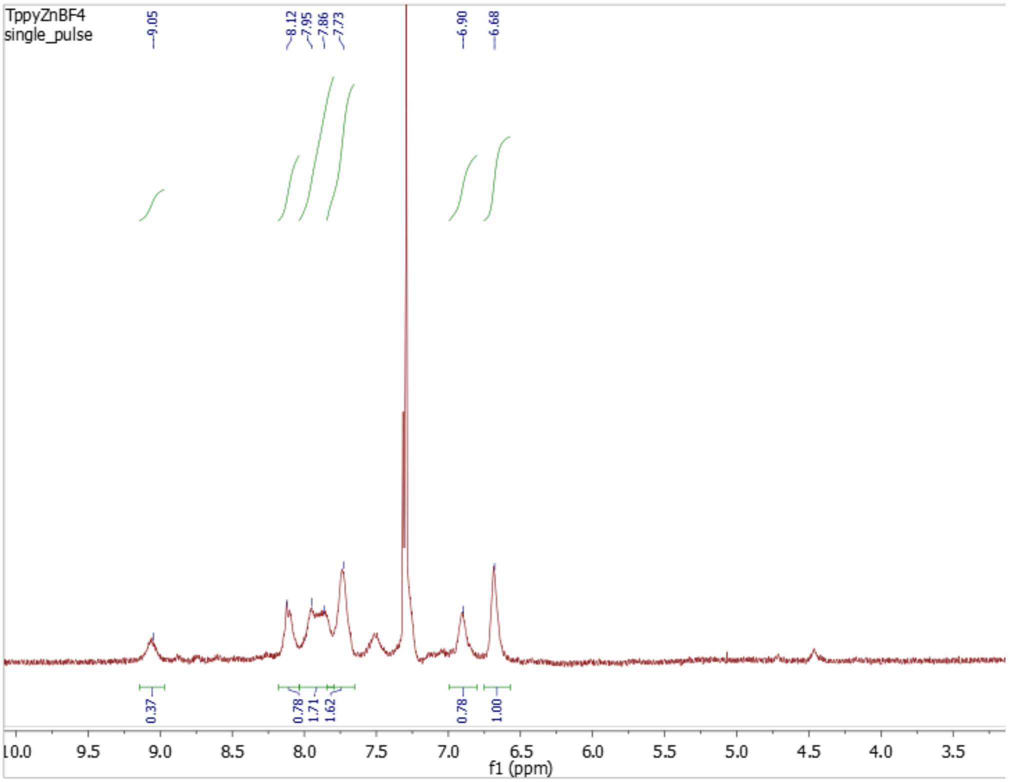

Figure S14. H^1-NMR spectrum for complex **2** in d-chloroform.

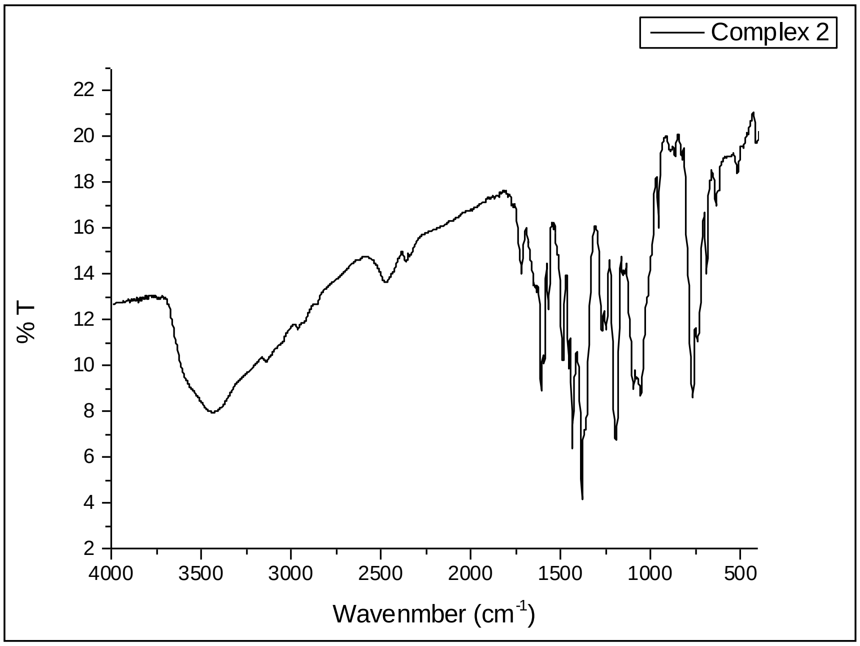

Figure S15. Solid IR spectra for complex **2**

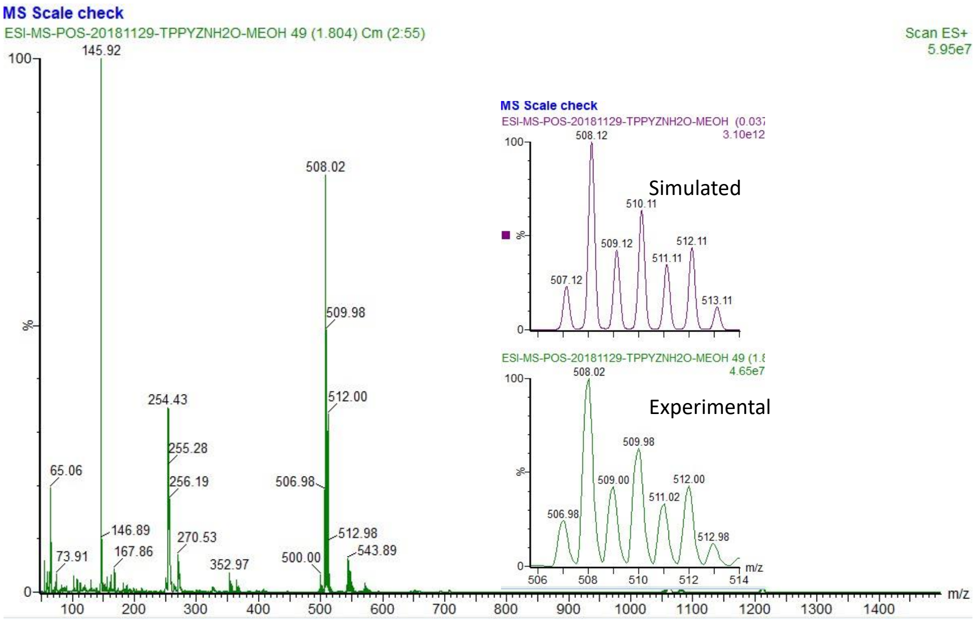

Figure S16. ESI-Mass spectra for complex **2**

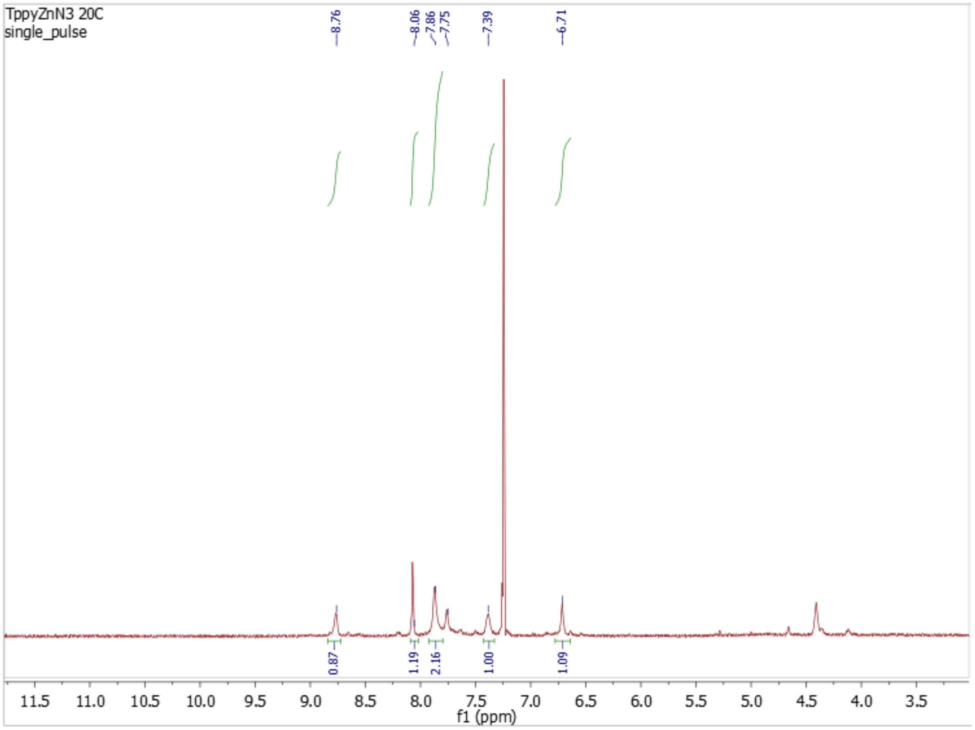

Figure S17. H[1]-NMR spectrum for complex **3** in d-chloroform.

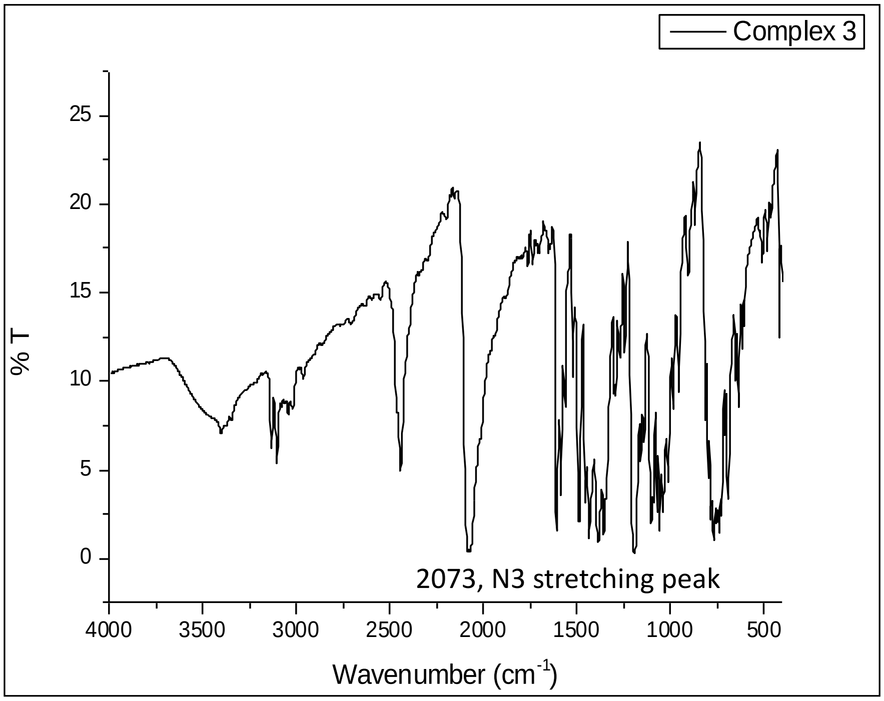

Figure S18. Solid IR spectra for complex **3**.

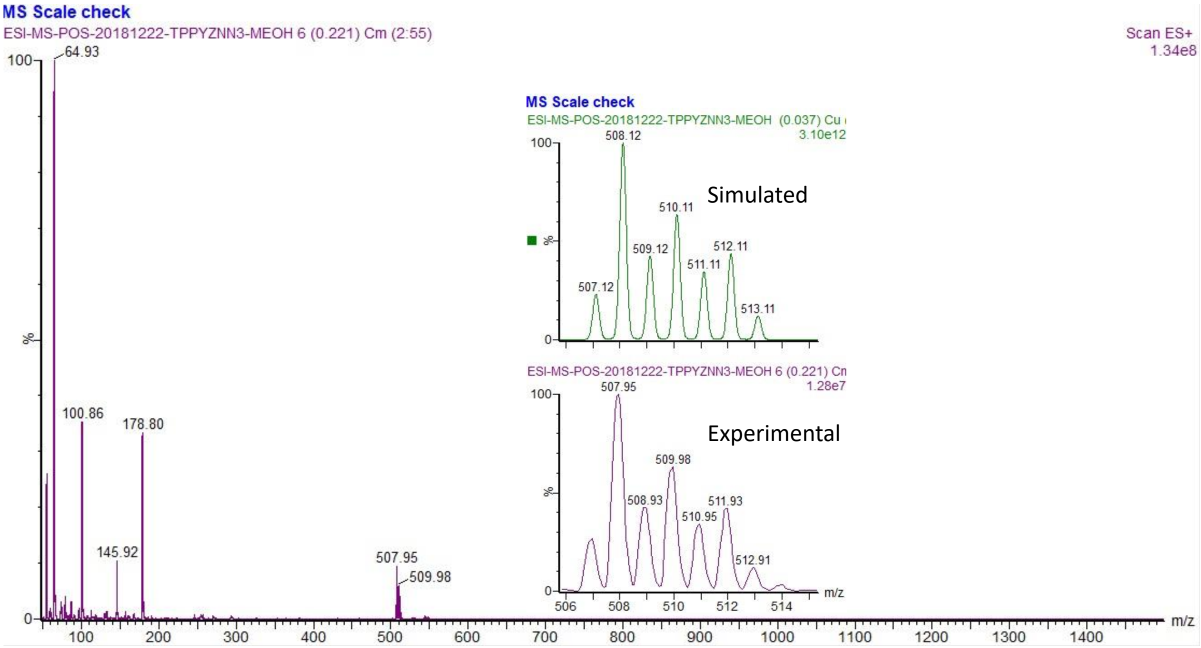

Figure S19. ESI-Mass spectra for complex **3**.

Table S1 Selected bond distances (Å) and angles (º) in complexes **1-3**

**Bond**	**1** (X= Cl)	**2** (X = OH_2)	**3** (X = N_3)
Zn–N_{pz}	2.148(3)	2.202(4)	2.221(5)
	2.075(3)	2.054(4)	2.075(5)
Zn–N_{py}	2.094(3)	2.035(4)	2.064(5)
	2.135(4)	2.180(4)	2.19–1(6)
Zn–X[a]	2.275(1)	1.954(3)	2.061(5)
N_{pz}–Zn–N_{py}	76.7(1)º	75.8(2)º	82.3(2)º
	138.8(4)º	146.8(2)º	140.9(2)º
N_{pz}–Zn–X	109.4(1)º	114.6(2)º	120.2(2)º
N_{py}–Zn–X	103.8(1)º	98.5(2)º	98.8(2)º

Table S2. Crystallographic data for complexes **1-3**

	**1**	**2**	**3**
Empirical formula	$C_{25}H_{21}N_{12}BZnCl_2$	$C_{25}H_{22}N_9B_2ZnF_4OCl_3$	$C_{26}H_{22}N_{10}BClZn$
Formula weight	636.62	733.86	586.17
Temperature	200(2)	200(2)	200(2)
Crystal system	Monoclinic	Triclinic	Monoclinic
Space group	*P* $2_1/c$	*P-1*	*P* $2_1/c$
a Å	11.1572(13)	8.0092(5)	9.152(2)
b Å	14.7713(18)	13.1053(10)	17.048(4)
c Å	16.9060(2)	15.3286(10)	17.156(4)
α °	90.00	109.987(2)	90.00
β °	102.228(4)	93.923(2)	100.883(6)
γ °	90.00	91.627(2)	90.00
R-factor	5.34	5.74	4.74

Z	4	2	4
Density (calc, g/cm^3)	1.553	1.618	1.481
Absorption coefficient(mm^{-1})	1.140	1.145	1.073
Crystal color, morphology	Prism/colorless	Prism/colorless	Prism/colorless
Crystal size(mm^3)	0.23 × 0.02 × 0.01	0.18 × 0.17 × 0.03	0.35 × 0.24 × 0.17
Refns meads/indep	51708/4815	35875/5316	17352/4582
Data/restrains/parameter	4815/0/369	5316/0/403	4582/0/353
GOF	1.099	1.024	1.112
R_{int}	0.1891	0.0685	0.0459
R_1[I>2σ](all data)	0.0589(0.1593)	0.0593(0.084)	0.0388(0.0532)
wR2 [I>2σ](all data)	0.1271(0.1900)	0.1374(0.1573)	0.0902(0.0956)
max peak/hole(e/Å^3)	0.815/-0.909	0.9665/-0.815	0.684/-0.663

Table S3. DNA binding constant (K_b), quenching constant (K_q) and apparent binding constant (K_{app}) values

Complex	K_b (M^{-1})	K_q(M^{-1})	K_{app} (M^{-1})
**1**	9.18×10^4	1.16×10^5	1.28×10^6
**2**	6.91×10^4	6.85×10^4	7.84×10^5
**3**	6.79×10^4	5.28×10^4	6.12×10^5
**Tp^{py} ligand**	3.18×10^4	------	-------

Table S4. Protein binding constant (K_b), quenching constant (K_q) and number of binding sites (n) values

Complex	K_b (M^{-1})	K_q(M^{-1})	n
**1**	6.90×10^5	2.57×10^5	1.37
**2**	6.45×10^5	1.78×10^5	1.23
**3**	5.37×10^4	1.19×10^5	1.07

Reference:

1. Lin, Y.-i.; Lang, S.A. Novel two step synthesis of pyrazoles and isoxazoles from aryl methyl ketones. *J. Heterocyclic Chem.* **1977**, *14*, 345-347.
2. Brunner, H.; Scheck, T. Asymmetrische Katalysen, 77[1] Neue optisch aktive Pyrazolderivate für die enantioselektive Katalyse. *Chem. Ber.* **1992**, *125*, 701-709.
3. Powell, H.R. X-ray data processing. *Biosci. Rep.* **2017**, *37*, BSR20170227.
4. Sheldrick, G. Crystal structure refinement with SHELXL. *Acta Crystallogr. C Struct. Chem.* **2015**, *71*, 3-8.
